# Titanium clip for lymph node marking in node-positive breast cancer: a reliable, cost-effective approach to targeted axillary dissection following neoadjuvant chemotherapy

**DOI:** 10.3332/ecancer.2026.2148

**Published:** 2026-06-16

**Authors:** Kanyadhara Lohita Krishna, Sasi Mouli, K S Bharat, Sunil Kalmath, Jismy Mathew, M S Sulakshana, B S Srinath

**Affiliations:** 1 Department of Surgical Oncology, Sri Shankara Cancer Hospital and Research Centre, 1st Cross Shankarapuram, Basavanagudi, Bengaluru, Karnataka 560004, India; 2 Department of Radiology, Sri Shankara Cancer Hospital and Research Centre, 1st Cross Shankarapuram, Basavanagudi, Bengaluru, Karnataka 560004, India; 3 Department of Pathology, Sri Shankara Cancer Hospital and Research Centre, 1st Cross Shankarapuram, Basavanagudi, Bengaluru, Karnataka 560004, India

**Keywords:** targeted axillary dissection, neoadjuvant chemotherapy, titanium clip

## Abstract

**Introduction:**

Targeted axillary dissection (TAD) is increasingly utilised in node-positive breast cancer patients who convert to node-negative status following neoadjuvant chemotherapy (NACT). TAD combines sentinel lymph node biopsy (SLNB) with removal of the previously marked metastatic node. Localisation of the marked node, however, remains one of the most technically challenging aspects of TAD and is a major barrier to its wider implementation. This study aimed to evaluate the clinical feasibility of using a low-cost titanium clip for lymph node (LN) marking and to determine the clipped node (CN) identification rate (IR) as well as its concordance with sentinel lymph nodes (SLNs).

**Materials and methods:**

This prospective feasibility study was conducted at Sri Shankara Cancer Hospital and Research Centre, Bangalore, between January 2024 and January 2025 after IEC approval. Patients with cN1, fine needle aspiration cytology/biopsy-proven nodal metastasis undergoing axillary LN clipping before NACT were included. The most suspicious LN was clipped using a coaxial system to deploy a titanium clip (Ligaclip, Ethicon Endo-Surgery, USA). Pre-operatively, the CN was localised using intra-operative ultrasound and direct skin marking. SLNB was performed using dual tracers: indocyanine green (ICG) combined with either methylene blue (MB) or radioisotope (RI). All patients subsequently underwent axillary lymph node dissection.

**Results:**

A total of 28 patients were included, with a mean age of 50.8 years. The CN IR was 100%, with an average CN size of 20 mm. In 92.5% of cases, the CN corresponded to the SLN. SLNB mapping success was 91.6% (11/12) with ICG + RI and 86.6% (13/15) with ICG + MB. A median of four LNs were retrieved during TAD. Nodal pathological complete response was achieved in 77.7% (21/27) of patients, more frequently among triple-negative (9/21) and Human epidermal growth factor receptor 2-positive (8/21) subtypes.

**Conclusion:**

The titanium clip demonstrated no migration and achieved 100% identification and retrieval of the CN, with high concordance to the sentinel node. These results establish clip-assisted TAD as a clinically feasible, reproducible and cost-effective approach that may facilitate wider adoption of TAD in node-positive breast cancer patients following NACT, particularly in resource-constrained settings.

## Introduction

Although axillary lymph node dissection (ALND) remains the gold standard for achieving locoregional control in node-positive breast cancer [1], sentinel lymph node biopsy (SLNB) is the established standard of care for axillary staging and management in node-negative patients [2, 3]. When compared with SLNB, ALND has higher rates of lymphedema, reduced strength and decreased range of movement [4]. The role of neoadjuvant chemotherapy (NACT) has expanded beyond locally advanced breast cancer to include selected cases of early-stage disease. Surgical management has evolved from routine total mastectomy to the possibility of breast-conserving surgery (BCS) in selected patients. Similarly, de-escalation of axillary surgery following NACT is now increasingly being adopted, with Nodal pathological responses ranging from ranging from 13% to 73% among various subtypes [5].

Evidence from previous studies have pushed us towards considering SLNB in post NACT setting. The challenges include lymphatic vessel fibrosis and non uniform tumour regression in metastatic lymph nodes (LNs), resulting in low detection rate (DR) and high false negative rates (FNRs) [6–8].

Targeted axillary dissection (TAD), which includes marking of positive LNs prior to NACT and intraoperative excision of the marked LNs along with Sentinel lymph nodes (SLNs) (more than three LNs retrival + dual tracer technique), has been shown to decrease the FNRs and is gaining popularity. This has been shown in a meta-analysis involving 2,217 patients [9].

Various markers are used for LN clipping and localisation to facilitate TAD. These markers can be broadly classified as radioactive or non-radioactive. Radioactive options include Iodine-125 seeds and Technetium-99 (Tc-99)-based tracers, while non-radioactive alternatives include magnetic or radiofrequency markers, tattoo ink, clip and wires, each with specific advantages and limitations depending on availability, cost and technical expertise [10]. Among the available options, products such as UltraClip BARD (INR 6000) and HydroMARK clip are frequently used. However, their limited availability and high costs restrict their routine use in many developing countries. Additionally, successful clip placement and subsequent localisation demand advanced technical skill, meticulous image-guidance and close coordination between radiology and surgical teams to ensure precise placement and reliable intraoperative retrieval.

To address the limitations associated with currently available methods, we describe an innovative, cost effective, one stage technique for axillary LN clipping utilising titanium Surgical Clip, which are conventionally employed to mark the tumour bed in BCS to facilitate accurate radiotherapy boost delivery.

This study presents a detailed account of the technical methodology and outcomes involved in LN clipping using a low-cost titanium clip and examines the feasibility of its identification within axillary LN following NACT in a clinical setting. It further aims to evaluate the identification rate (IR) of the clipped node (CN) and its concordance with SLNs, thereby assessing the accuracy and potential utility of this low-cost technique in facilitating TAD following neoadjuvant therapy.

## Material and methods

This single-institution prospective feasibility study included patients who underwent LN clipping between January 2024 and January 2025. The study was approved by the Institutional Ethics Committee of Sri Shankara Cancer Hospital and Research Centre, Bangalore (SSCHRC/IEC19/160). All patients provided written informed consent. We designed the study to determine the clinical feasibility of using a low-cost titanium clip for LN marking in patients with node-positive breast cancer (only cN1).

Inclusion criteria

- Clinically and radiologically T1-3N1.
- All breast cancer subtypes.
- N1 – according to the AJCC staging system cN1 is defined as disease in movable axillary LNs.
- Histologically proven invasive breast carcinomas and histologically confirmed LN metastasis diagnosed with fine needle aspiration cytology/aspiration biopsy (FNAB) or core-needle biopsy (CNB).

Exclusion criteria

- Patients who have received any therapy (chemotherapy, endocrine therapy and radiotherapy) prior to NACT.
- Distant metastasis, male breast carcinoma, inflammatory breast carcinoma, bilateral breast carcinoma and pregnancy.
- Patients with four or more involved LNs.
- Hypersensitivity to fluorescent dye or allergy to blue dye or metal.

The DR of the CN was defined as the proportion of patients in whom the clipped LN was successfully identified and retrieved intraoperatively. Secondary endpoints included the number of LNs retrieved during TAD and the concordance between the CN and SLN.

Clinicopathologic data were prospectively obtained from source documents within the electronic medical record, including clinic notes and reports from the radiology and pathology departments.

### Nodal ultrasound and titanium clip placement

**Figure 1.**
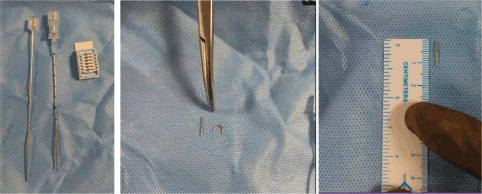
(a): Co-axial system and titanium clip; (b): titanium clip straightened out; (c): size of titanium clip.

**Figure 2.**
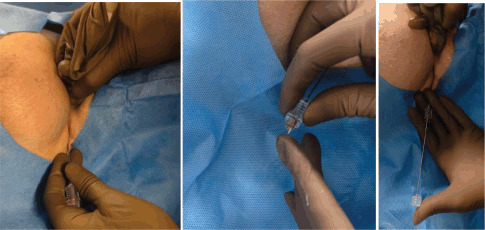
(a): Introduction of coaxial system; (b): titanium clip is inserted into the coaxial system; (c): Blunt-end plunger being used to displace the titanium clip within the coaxial into the LN.

In accordance with institutional protocol, ultrasonography was carried out to evaluate the ipsilateral regional lymphatic basins, including the axillary, infraclavicular and internal mammary regions, in all patients diagnosed with breast cancer. Suspicious axillary LNs were characterised according to the Bedi classification [11], based on sonographic findings such as asymmetric cortical thickening, loss or displacement of the fatty hilum, hilar compression, cortical irregularity and margin distortion (Figures 1 and 2).

Patients underwent ultrasound-guided FNAB of the axillary LN using a 21-gauge needle. Following cytologic or histologic confirmation of metastasis, sterile titanium clip costing INR600 per clip (LigaClip MCA MSM20; Ethicon Endo-Surgery, USA) were straightened and introduced into the cortex of the sampled LN under ultrasound guidance. A coaxial system was used to accurately deliver and deploy the clip within the targeted node. The correct position of the tip of the coaxial system within the desired portion of the LN was confirmed sonographically. Post-procedure verification of clip placement was performed in all cases using both ultrasound and mammography.

### Radiological localisation

Following NACT, a response mammogram along with ultrasound was repeated to confirm clip location, assess for displacement and evaluate LN response.

### Surgical procedure

Prior to surgery, the clipped LN was re-visualised under ultrasound guidance, and its position was marked on the skin to facilitate intra-operative identification. Secondary localisation procedures were not required, as direct skin marking and intraoperative ultrasound were sufficient for accurate CN identification.

SLN procedure (either of the two techniques was used in combination)

- Preoperative lymphoscintigraphy was performed following subdermal or peritumoural injection of 15–20 MBq of Tc-99on the day of surgery. Intraoperatively, a handheld gamma probe was used to detect areas of maximum radioactivity within the axilla. All LNs demonstrating a radioactive count greater than 10% of the *ex vivo* activity of the hottest SLN were identified and excised.
- 3 mL of Patent Blue dye will be injected subcutaneously subareolar at 3 and 9 o’clock positions, followed by massage to facilitate the movement of dye
- 3 mL of AUROGREEN^®^ 25 mg/10 mL will be injected subcutaneously at 6 and 12 o’clock positions, followed by massage to facilitate the movement of dye. Movement of indocyanine green (ICG) in the lymphatic ducts will be visualised through Irillic.nm fluorescence imaging (Irillic, India)

An incision was made along the anterior axillary fold, and the clipped LN was identified (by direct skin marking and intra-operative ultrasound) along with the SLN. Intraoperative specimen radiography was performed in all cases to confirm the presence of the clip within the excised LN. Following TAD, completion of the axillary dissection was carried out according to standard surgical principles. Level III LN dissection was performed only when intraoperative findings or preoperative imaging suggested metastasis; in all other cases, axillary dissection was confined to levels I and II.

In cases where TAD could not be successfully completed, an ALND was performed directly. Such cases were included in the analysis of DRs but excluded from FNR calculations.

### Pathological handling

On arrival at the pathology laboratory, the specimen underwent accession and gross pathological examination. All removed TAD LNs underwent routine Histopathological evaluation. Formalin-fixed, paraffin-embedded LN tissue blocks were sectioned at 5 μm thickness and stained with hematoxylin and eosin. The LN containing the clip was serially sectioned and processed separately, following the same routine histopathological protocol used for other LNs. Each LN retrieved in the TAD specimen was examined individually, and its morphologic characteristics were documented. Immunohistochemical staining was performed in cases where metastatic involvement was equivocal on routine staining to identify isolated tumour cells. LNs demonstrating macrometastases, micrometastases or isolated tumour cells were classified as positive. Pathologic complete nodal response (ypN0) was defined as the absence of viable tumour cells in all examined LNs.

Continuous variables were recorded as medians with range and categorical variables as numbers and proportions.

## Results

Between January 2024 and January 2025, a total of 654 patients were diagnosed with invasive breast cancer at our institution. Of these, 240 patients received NACT and 28 patients who met the predefined inclusion criteria were enrolled in the study.

Clinicopathologic characteristics of the patients are summarised in Table 1.

The median age was 52 years (range, 38–70), and the mean BMI was 27.7 ± 2.0 kg/m². 42.8% were pre-menopausal. The mean tumour size at diagnosis was 24.0 ± 2.87 mm. The mean long- and short-axis diameters of the clipped LN were 15.9 ± 2.67 and 10.0 ± 1.69 mm, respectively. Most patients presented with cT2 disease (82.1%), and half had two abnormal nodes on baseline ultrasound (50%). All tumours were invasive ductal carcinomas, with the majority being grade 2 (64.2%). Molecular subtypes were evenly distributed, with triple-negative breast cancer representing 35.7% of cases. Neoadjuvant regimens included anthracycline-taxane combinations (39.2%), taxane-carboplatin with dual Human epidermal growth factor receptor 2 (HER2)-targeted therapy (21.4%), taxane-carboplatin with single HER2-targeted therapy (21.4%) and anthracycline-based regimens alone (17.8%). All (HER2)-positive patients received HER2-directed therapy. The mean NACT duration was 4 months (3–6 months). Based on RECIST criteria, 67.8% of patients achieved a clinical complete response, and 71.4% were ycN0 at post-NACT axillary clinical staging. BCS was performed in 71.4% of cases and breast pCR was observed in 46.4%. Pathologically, 75% of patients were ypN0, and total pCR was achieved in 46.4%.

The position of the clip in the axilla was confirmed by mammogram and ultrasound in all patients. All patients had successful radiologic localisation and skin marking of the clip-containing LN pre-operatively. A median of four LNs were removed during TAD. The CN was successfully retrieved as a part of TAD in 28/28 patients, resulting in an overall IR of 100%. This was radiologically confirmed by specimen X-ray.

SLN mapping was successful in 89.2% (25/28) of patients. When stratified by mapping technique, the IR was 91.6% (11/12) with the combination of ICG and radioisotope (RI) and 87.5% (14/16) with ICG and methylene blue (MB). In 92% (23/25) of patients in whom SLNs were identified, the CN was also an SLN, indicating a high concordance rate (Table 2).

Residual nodal disease was present in 25% (7/28) of patients. Among these, metastasis was detected in the CN in five cases and by SLNB in six cases. When CN and SLNB were combined (TAD), six of seven patients with residual disease were correctly identified as node-positive. The mean number of LNs retrieved during ALND was 13.3 (range, 2–24), with additional axillary LN positivity observed in four patients.

## Discussion

Management of the axilla in breast cancer patients presenting with clinically node-positive disease who convert to ycN0 following NACT continues to evolve. Although ALND has long been the standard approach, it is associated with considerable morbidity, including lymphedema, sensory deficits and shoulder dysfunction, without conferring additional oncologic benefit in all patients. In contrast, TAD – which entails the excision of the previously marked metastatic node along with SLNs – has emerged as an oncologically safe and less invasive technique, with a 5-year axillary recurrence rate of 1%, that preserves staging accuracy while minimising surgical [12].

In most cases, a two-stage marking approach is necessary, with initial clip placement before NACT and a secondary localisation method, such as wire/ink/radioactive or magnetic seed/radar markers post NACT. However, image-guided retrieval becomes increasingly challenging as the interval between the initial clip placement and subsequent localisation lengthens, particularly in cases with marked radiological or pathological response to systemic therapy.

Previous prospective studies have reported variable outcomes regarding ultrasound visibility of CNs after neoadjuvant therapy, with rates ranging from 72% to 83.3% [13]. Another study evaluating I-125 seed localisation under ultrasound guidance reported a success rate of only 76%, with unsuccessful placement in 24% of cases when the seed was positioned more than 1 cm from the target, highlighting the challenges of seed-based localisation [14, 15]. In contrast, clip-based wire localisation demonstrated lower overall performance, with ultrasound visibility in 83.3% of cases and an IR of only 70.8% [13]. Kanesalingam *et al* [16] faced challenges of clip visualisation post NACT and observed IR of 78%.

The multicenter pooled analysis of 17 studies, including more than 1,300 patients, confirmed the feasibility of TAD, with localisation and retrieval rates of 97% and 99%, respectively [17]. Nevertheless, each technique studied carried limitations: radioactive seeds required strict regulatory oversight and specialised handling; Magseed and radar reflectors, though highly accurate, were constrained by cost and probe dependence; carbon tattooing showed inconsistent visibility post-NACT; and wire localisation was prone to migration, same-day placement requirements and patient discomfort. In the present study, titanium clip demonstrated no migration and achieved 100% success in both ultrasound visualisation and intraoperative retrieval of the CN

Kanesalingam *et al* [16] reported that the CN did not correspond to the SLN in 14% of patients. Similarly, García-Novoa *et al* [18] demonstrated concordance between the CN and SLN in 80% of cases. More recently, Moore *et al* [19] from MD Anderson evaluated 680 patients undergoing TAD and found the CN to be an SLN in 90% of cases. On multivariate analysis, the presence of more than three suspicious nodes on ultrasound and larger primary tumour size were significantly associated with the CN not being an SLN. In our present study, the CN corresponded to the SLN in 92.5% of patients, supporting a high concordance rate. These findings suggest that titanium clip not only provide reliable localisation but may also enhance the accuracy of SLN identification, with performance comparable or superior to previously reported series.

A significant challenge in implementing TAD globally lies in the limited availability of localisation seeds and devices. In countries such as India and many other low- and middle-income regions, the adoption of radioactive seed localisation is constrained by complex safety regulations and stringent national radiation protection laws. Similarly, magnetic and radar-based systems, though effective, are prohibitively expensive and not widely accessible. In contrast, titanium clip offer a simple, low-cost and universally available alternative that avoids these regulatory and logistical barriers. Secondary localisation was not required in any case. The titanium clip was consistently visible on intraoperative ultrasonography due to acoustic shadowing. Real-time intraoperative ultrasound identified the CN, followed by direct skin marking. In addition, clip-induced fibrosis resulted in palpable nodal enlargement (average 20 mm) in most cases. The combination of ultrasound visualisation, palpability and dual-tracer mapping facilitated the reliable excision of the CN.

The economic implications of marker selection are particularly relevant in low- and middle-income countries, where the cost of specialised clip systems can limit widespread adoption of TAD. The UltraClip (BARD) costs approximately INR 6,000 per clip, whereas the standard surgical titanium Clip, widely available in most operating rooms, costs about INR 600. This nearly ten-fold cost difference highlights a significant advantage of the titanium Clip, making it a more practical and sustainable option for routine use. By offering equivalent feasibility in intraoperative detection and retrieval at a fraction of the cost, titanium clips provide an accessible alternative that may enable broader implementation of TAD in resource-constrained settings.

To our knowledge, data on the histological size of CNs after NACT in the TAD setting are scarce. Most published studies have focused on localisation and retrieval rates, with limited information on nodal dimensions post-treatment. The SMART trial [20] reported ultrasound-based measurements of CNs after NACT, showing that 42.9% measured <5 mm, 42.9% between 5 and 9 mm and only 14.2% >9 mm [20]. These findings highlight that CNs often shrink substantially and may become challenging to visualise or retrieve. However, no published studies have specifically reported on the histological size of CNs following NACT. Titanium clip induced localised fibrosis, which causes the surrounding nodal tissue to encapsulate the marker, maintaining an average CN size of approximately 20 mm even after NACT. This consistent enlargement facilitated reliable ultrasound visibility and palpability, simplifying intraoperative localisation and obviating the need for secondary localisation procedures.

Taken together, our findings support the role of titanium clip as a practical and oncologically safe localisation method in the setting of TAD. By eliminating the need for a second localisation procedure – which otherwise entails additional cost, an extra invasive intervention and increased reliance on interventional radiology time.

## Conclusion

Titanium Clip-based TAD is clinically feasible, reproducible and cost-effective, with minimal technical complexity. By eliminating the need for secondary localisation procedures, this approach reduces resource utilisation. Furthermore, the high concordance between CN and sentinel node reinforces its oncologic reliability. Adoption of this technique has the potential to expand the applicability of TAD globally, allowing more patients to benefit from less invasive axillary management.

## Conflicts of interest

The authors declare that the research was conducted in the absence of any commercial or financial relationships that could be constructed as a potential conflicts of interest.

## Funding

No.

## Figures and Tables

**Figure 3.**
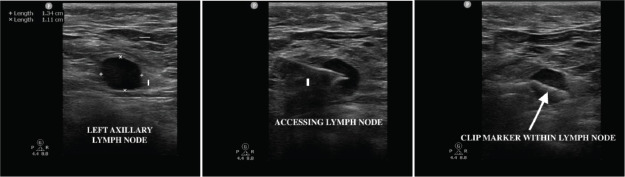
(a): Axillary LN with loss of fatty hilum; (b): introduction of coaxial system into LN; (c): titanium clip within the node.

**Figure 4.**
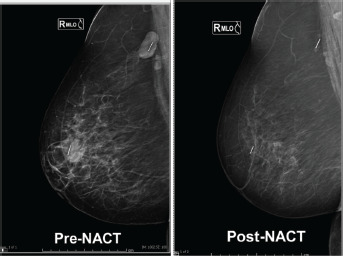
Pre-and post-NACT mammographic images demonstrating persistent visualization of the Ligaclip within the axillary LN without migration following NACT.

**Figure 5.**
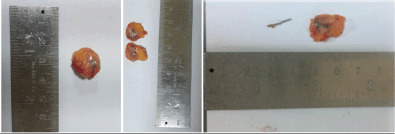
Gross specimen of the excised clipped LN with cut section showing the retrieved titanium clip within the nodal specimen.

**Table 1.** Clinicopathological features.

Characteristics	All patients (n = 28)
Age, mean(range) years	52 (8–70)
Menopausal status	
Pre-menopausal	12 (42.8%)
Post -menopausal	16 (57.1%)
BMI, kg/m2	27.7 ± 2.0
Tumour size at diagnosis, mm	24.0 ± 2.87 mm
Long diameter of CLN at diagnosis, mm	15.9 ± 2.67 mm
Short diameter of CLN at diagnosis, mm	10.0 ± 1.69 mm
Pretreatment clinical T classification	
T1	5 (17.9%)
T2	23 (82.1%)
Number of abnormal LNs based on ultrasound at diagnosis	
1	9 (32.1%)
2	14 (50%)
3	5 (17.9%)
Histological tumour type	
Ductal carcinoma	28 (100%)
Tumour grade	
1	0
2	18 (64.2%)
3	10 (35.8%)
Molecular subtype	
ER+/HER2-	6 (21.4%)
ER+/HER2+	6 (21.4%)
ER-/HER2+	6 (21.4%)
ER-/HER2-	10 (35.7%)
NACT	
Anthracycline + Taxane	11 (39.2%)
Anthracycline only	5 (17.8%)
Taxane + carboplatin + dual target	6 (21.4%)
Taxane + carboplatin + single target	6 (21.4%)
RECIST- based treatment response	
CR	19 (67.8%)
PR	9 (32.1%)
Clinical axillary staging after NAC	
ycN0	20 (71.4%)
ycN+	8 (28.5%)
Breast surgery	
Mastectomy	8 (28.5%)
Lumpectomy	20 (71.4%)
Breast PCR	13 (46.4%)
Pathological axillary staging after NAC	
ypN0	21 (75%)
ypN1	5 (17.8%)
ypN2	2 (7.1%)
ypN3	0
Total PCR	
Yes	13 (46.4%)
No	15 (53.5%)

**Table 2.** CN IR and concordance with SLN.

Parameter	Result
Clip position confirmation	100% (28/28) by mammography and ultrasound
Preoperative localization & skin marking	Successful in 100% (28/28)
Mean number of nodes removed (TAD)	4.3 ± 2.24
CN retrieval rate	100% (28/28), confirmed by specimen X-ray
SLN mapping success (Overall)	89.2% (25/28)
SLN IR by technique	91.6% (11/12) with ICG + RI 87.5% (14/16) with ICG + MB
Concordance of CN with SLN	92% (23/25)
